# *Bacillus thuringiensis* Sublethal Concentration Effectively Alter Cytogenetic Response of *Spodoptera frugiperda* Larvae

**DOI:** 10.1007/s13744-026-01384-9

**Published:** 2026-04-16

**Authors:** Marian Malak, Mourad Shonouda

**Affiliations:** 1 Biological and Geological Sciences Dept, Faculty of Education, Alexandria Univ, Alexandria, Egypt; 2 Zoology Dept, Faculty of Science, Alexandria Univ, Alexandria, Egypt

**Keywords:** *Spodoptera frugiperda*, Btk, DNMT, Midgut, Histopathological, Apoptosis

## Abstract

**Abstract:**

*Bacillus thuringiensis* (Bt)–based microbial insecticides are crucial for controlling the maize pest, *Spodoptera frugiperda* (Smith) (Lepidoptera: Noctuidae). While the insecticidal efficacy of Bt has been well-documented, its sublethal cytogenetic toxicity on midgut cells remains less explored. This study aimed to evaluate the sublethal concentration toxicity of *Bacillus thuringiensis*, var. *Kurstaki* (Btk), on survival percentage, genotoxicity, histopathology, and ultrastructure of the midgut cells of *S. frugiperda* larvae. By calculating lethal concentration (LC) values (LC_25_, LC_50_, LC_75_, and LC_90_) in the laboratory, the insecticidal efficacy was ascertained. The Btk lethal concentration was toxic to third-instar *S. frugiperda* larvae after 48 h, with an LC_50_ of 109 µg/100 mL (106–112 µg/100 mL). Subsequently, the results demonstrated that LC_25_ resulted in a 22% reduction in larval survival percentages, so larval midgut tissues treated with the sublethal concentration (LC_25_) were isolated for further cytogenetic analysis. Consequently, Btk treatment markedly disrupted midgut cells, inducing micronuclei (MN) formation and reducing *SfDnmt2* mRNA gene expression considerably. Furthermore, cytotoxicity has been shown in *S. frugiperda* midgut epithelial cells by cellular disorganization, fragmentation and protrusion, peritrophic matrix rupture, and microvilli degeneration. Btk sublethal concentration also induced cytotoxicity by causing apoptosis and necrosis in *S. frugiperda* midgut cells. These findings suggest that sublethal concentrations of Bt induce significant cytogenetic stress, which may complement the role of Bt-based biopesticides in integrated pest management strategies as a promising, environmentally safe method for decreasing the survival rate and controlling the pest *S. frugiperda*.

**Graphical Abstract:**

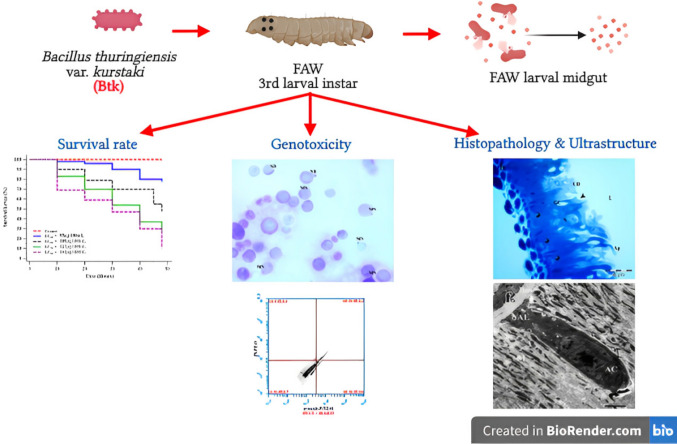

**Supplementary Information:**

The online version contains supplementary material available at 10.1007/s13744-026-01384-9.

## Highlights

• Bacillus thuringiensis is a Gram-positive bacterium used as an effective microbial insecticide

• The survival percentage of Spodoptera frugiperda larvae reduced after LC25 of Btk treatment

• Apoptosis and necrosis processes were activated after treatment with LC25 of Btk

• LC25 of Btk induced pronounced histological and ultrastructural alterations of midgut cells

**Supplementary Information:**

The online version contains supplementary material available at 10.1007/s13744-026-01384-9.

## Introduction

Fall armyworm (FAW), *Spodoptera frugiperda* (Smith) (Lepidoptera: Noctuidae), possesses a notable capability for causing damage to a wide variety of cultivated plants worldwide. Its larvae are recognized as a major destructive pest of corn and cotton, causing significant economic losses (Montezano et al. 2018). The infestation of agricultural areas with *S. frugiperda* has led to frequent use of insecticides, increased control expenses, and the potential for pest resistance, harmed the environment, and posed risks to farmers (dos Santos et al. 2009).

Furthermore, prolonged application of chemical insecticides could harm non-target organisms, heighten environmental pollution, and potentially lead to food safety issues (Casida and Durkin 2017). Currently, a significant challenge faced by researchers is insect pest resistance to pesticides. As a result, microbial insecticides that are both safe and eco-friendly are gaining popularity (Yang et al. 2023). They exhibit great potential as an alternative to chemical insecticides. The benefits of using microbial insecticides are their sustainable manufacturing and easy mass production in a laboratory (Ayilara et al. 2023).

*Bacillus thuringiensis* (Bt) is one of the most widely applied microbial insecticides. Bt is a Gram-positive bacterium that produces toxic parasporal crystals and endospores and can manage agricultural pests (Jouzani et al. 2017). Following feeding on Bt, the crystals are solubilized by the action of the alkaline pH in the larval midgut, releasing delta-endotoxin (Xiao and Wu 2019). Delta-endotoxin is a bacterial toxin belonging to the pore-forming toxin class. Therefore, Bt has evolved strategies to inhibit host immune responses, thereby increasing the chances of survival and reproduction in the insect host. For example, Bt delta-endotoxins can promote the formation of micronuclei (MN) within cells, initiating the apoptosis process (Grisolia et al. 2009; Utani et al. 2010). Apoptosis plays a crucial role in maintaining the homeostasis and growth of organisms and has been identified as a cellular response following exposure to Bt delta-endotoxins (Zou et al. 2024). Furthermore, DNA methylation promotes apoptosis, which is correlated with Bt infection (Hou et al. 2020; Kausar et al. 2021). DNA methylation occurs when a methyl group is covalently transferred to the C-5 position of the cytosine (CG sites) of DNA. It plays various roles in physiological processes by disturbing chromatin stability (Li et al. 2022), stress resistance tolerance (Chen et al. 2020), and gene expression performance (Zhang et al. 2018). Interestingly, DNA methylation is mediated by DNA methyltransferases (DNMTs), which are evolutionarily conserved in insects (Bewick et al. 2017). In response to bacterial infection, the expression of the DNMT gene may alter, which can affect the pathogenic bacteria’s reproduction rates and larval mortality (Baradaran et al. 2019; Lai and Wang 2025). Similarly, Bt creates pores in the plasma membrane, disrupting cell permeability and the cellular integrity of insect midgut cells (Soberón et al. 2018). Consequently, the larvae die due to starvation or septicemia, or they live but suffer sublethal impacts such as reduced feeding, growth, and development (Pardo-López et al. 2013). The effectiveness of Bt as a microbial insecticide was influenced by its interaction with the midgut of larvae (Polenogova et al. 2021). The goblet cells in the insect midgut are considered the main physiochemical barrier that helps maintain the balance of ions, and digestive cells produce enzymes for digestion and absorb nutrients (Terra et al. 2006; Gabarty et al. 2021). Alterations in goblet and digestive cells have been observed in the midgut of *Spodoptera litura*, and *Spodoptera littoralis* following exposure to Bt, respectively (Pandey et al. 2009; Khalil et al. 2021). Moreover, infection by *Bacillus thuringiensis* var. *kurstaki* strain (Btk) induces stress in insect pests; therefore, Btk is commonly used for the control of lepidopteran larvae such as *S. frugiperda* (Sanahuja et al. 2011; Moustafa et al. 2024). *Spodorptera frugiperda* larvae exhibit high mortality when exposed to lethal concentrations of toxic pesticides without addressing how these toxins affect insect cells. Although the formation of pores and subsequent histopathological damage in lepidopteran midguts are well-established primary effects, the secondary cytogenetic effects of Bt toxins in *S. frugiperda* are still poorly understood. Accordingly, the present study aimed to evaluate the consequences of treating economically destructive third-instar *S. frugiperda* larvae with the sublethal Btk concentration (LC_25_) on survival percentage, genotoxicity, histopathology, and ultrastructure of midgut cells.

## Materials and methods

### Insect rearing

Larvae of different *S. frugiperda* instars were supplied by the Plant Protection Research Institute, Agricultural Research Center (ARC). The larvae were fed fresh castor leaves (*Ricinus communis*) daily, which are readily available and used extensively for rearing *S. frugiperda* (Nandhini et al. 2023). The larvae were reared in containers covered with fine muslin, measuring 25 cm in length, 15 cm in width, and 12 cm in height. From the third instar until pupation, larvae were kept individually and separately to prevent cannibalism. Pupae were examined and collected daily. Adults were provided with a 10% sugar solution upon their emergence and fresh castor leaves for egg deposition within the mating jars. Egg masses were collected daily and stored in separate containers. They were reared under laboratory conditions for 10 generations at favorable growth conditions (temperature of 27 ± 1°C, relative humidity of 70 ± 5%, and photoperiod L12:D12).

### Microbial insecticide concentrations and concentration-mortality bioassay

Protecto® is a commercial Btk formulation obtained from the Bioinsecticide Production Unit (ARC). Btk produces crystalline delta-endotoxins during its stationary growth phase. Protecto® contains 9.4% wettable powder (W.P.) as an active ingredient. Nine concentrations were prepared from the wettable powder, ranging from 85 to 150 µg, dissolved in 100 mL of double-distilled water (ddH_2_O), and used in a preliminary study to determine the lethal and sublethal concentrations (Supplemental Table 1). Lethal concentrations that resulted in larval mortality of 25% (LC_25_), 50% (LC_50_), 75% (LC_75_), and 90% (LC_90_) were bio-assayed to evaluate the toxicity under laboratory conditions. The concentration-response curve was used to determine lethal and sublethal concentrations. Subsequently, a bioassay experiment was conducted using Btk at a sublethal concentration (LC_25_). All bioassay tests of lethal and sublethal concentrations were done according to the leaf dip method (Naeem-Ullah et al. 2020). Fresh, equal-sized castor leaves were cleaned under running water before being dried in the laboratory. Afterwards, they were soaked in lethal Btk concentrations (group 1) for 60 s before they were left to air dry in the laboratory for 1 h. After a 4-h starvation period, 20 third-instar larvae were placed in a Petri dish and provided with the Btk-treated castor leaves for 24 h. Subsequently, they were fed untreated castor leaves until pupation. As a control group (2), 20 additional third-instar larvae were fed on clean castor leaves under the same conditions. Five replicates were conducted for both the control and treated groups.

### Time‑mortality bioassay

The *S. frugiperda* larvae were separated into Petri dishes and exposed to lethal concentrations of Btk corresponding to the LC_25_/48 h, LC_50_/48 h, LC_75_/48 h, and LC_90_/48 h. The control group was treated with ddH_2_O. The number of surviving larvae was recorded every 6 h for 48 h. Each Btk lethal concentration was tested on five replicates of 20 individual larvae.

### Sample preparation

After 48 h, ten larvae from either the control or treated dishes with LC_25_ of Btk were dissected and examined to evaluate Btk genotoxicity and the *S. frugiperda* DNA methyl transferase 2 (*SfDnmt2*) gene expression level. The midgut tissues were rinsed with cold phosphate-buffered saline (PBS) and then placed in 1.5-mL centrifuge tubes for each replicate. Three biological replicates were conducted, and all samples were promptly frozen in liquid nitrogen and stored at −80°C for further genotoxicity and molecular analysis (Li et al. 2019).

### Genotoxicity assay

#### Micronucleus test (MN)

The MN test was utilized to evaluate the genotoxic effects of Btk (LC_25_) on the midgut of third-instar *S. frugiperda* larvae, for both control and treated groups, following Fenech’s (2007) protocol. After extracting the tissues, the samples were submerged in a saline solution and subjected to hypotonic treatment using tap water for genotoxicity evaluation. The tissue samples were placed in a colchicine solution for 1 h to achieve large mitotic metaphases. A LEICA DM6 M LIBS microscope, at ×100 magnification, was used to assess the MN. The number of cells was evaluated according to the MN index’s variation. Micronuclei formation was measured and scored in 1000 cells. Consistent with Ahmadi et al. (2015), a minimum of 1000 binucleate cells were examined per slide to determine the presence or absence of MN. The experiments were repeated three times.

#### Annexin V-FITC assay

To determine if LC_25_ of Btk impacted apoptosis and necrosis in *S. frugiperda* midgut cells, the analysis was conducted with the Annexin V-FITC assay kit (Sigma-Aldrich, Germany). To summarize, PBS was utilized for homogenizing midgut tissues from control and treated larvae, as mentioned in studies by Peng et al. (2001), Yedjou et al. (2010), and Dabour et al. (2019). Following this, a combination of 195 µL of cell suspension binding buffer (50 mL binding buffer and 150 mL distilled water) was mixed with 5 µL of Annexin V-FITC, stirred, and afterward incubated in the dark for approximately 10 min. Three duplicate slides were prepared, with 100 cells evaluated per slide. After washing and suspending the cells in 190 µL of binding buffer, 10 µL of propidium iodide (PI) solution was added. Following placement on ice, the cells underwent flow cytometry analysis using equipment (Becton Dickinson, Franklin Lakes, NJ, USA). The collected results were analyzed using Becton Dickinson’s CellQuest software. Three biological replicates were allocated for the treatments.

#### *S. frugiperda* DNA methyl transferase 2 (*SfDnmt2*) gene expression level

##### Total RNA extraction

The standard TRIzol Reagent extraction procedure (Cat#15596026, Invitrogen, Germany) was used to extract total RNA from midgut tissues of 10 larvae per replicate from both control and Btk-treated insects. This procedure followed the manufacturer's instructions. All RNA was processed with 1 unit of RQ1 RNAse-free DNAse from Invitrogen, Germany, to eliminate any DNA impurities and then was suspended in diethylpyrocarbonate-treated water. The total RNA purity was evaluated with an OPTIZEN spectrophotometer by measuring the 260/280 nm ratio (ranging from 1.8 to 2.1). Moreover, the integrity was confirmed through agarose gel electrophoresis. Reverse transcription of aliquots was conducted immediately using the RevertAid™ First Strand cDNA Synthesis Kit (Thermo Fisher Scientific, Waltham, MA, USA) with the supplied oligo-dTs for reverse transcription (RT). The *RPL10* (60S ribosomal protein L10) gene was selected as a candidate reference gene to assess expression stability (Boaventura et al. 2019; Han et al. 2021).

##### Real-time polymerase chain reaction (RT-qPCR)

RT-qPCR was used to measure the relative expression level of *SfDnmt2*. The *S. frugiperda*’s cDNA copy number was determined using the StepOne™ Real-Time PCR System from Applied Biosystems (Thermo Fisher Scientific, Waltham, MA, USA). PCR reactions were prepared with 25 µL mixes comprising 12.5 µL 1× SYBR® Premix Ex Taq™ (TaKaRa, Biotech. Co. Ltd.), 0.5 µL 0.2 µM primers in the forward direction, 0.5 µL 0.2 µM primer in the reverse direction, 6.5 µL distilled water, and 5 µL cDNA template. The reaction program was divided into 3 stages. The initial phase occurred at 95.0°C for 3 min. The next phase involved 40 cycles, with each cycle divided into 3 steps: (a) 15 s at 95.0°C; (b) 30 s at 60.0°C (for *SfDnmt2* gene) and 30 s at 57.0°C (for *RPL10* gene); and (c) 30 s at 72.0°C. Melting curve analysis was performed over 71 cycles, beginning at 60.0°C and increasing by 0.5°C every 10 s until reaching 95.0°C. The specific primer sequences for the examined gene were created with Primer-3 software (Table 1). Melting curves were analyzed to verify the presence of unique PCR products. The relative quantification method was used to calculate mRNA expression levels using the 2^−ΔΔCT^ method described by Schulz et al. (2022). The experiment was repeated three times with complete independence.

**Table 1:** Primer’s sequence used for RT-qPCR

Gene	Primer sequences	GenBank (Accession Number)
*SfDnmt2*	F: 5′-GCACTGTGCATGGAAAGAATC-3′	A0A2H1VE33
	R: 5′**-**TGGCATGGCGGTGACATAAG-3′	
*RPL10*	F: 5′-TGGGTAAGAAGAAGGCTACG-3′	A0A2H1VRE1
	R: 5′**-**TGTTGATGCGGATGACAT-3′	

### Histopathological examination of the midgut

Five larvae from the control or Btk-treated (LC_25_) were also chosen to study histological and ultrastructural changes in the midgut after 48 h of treatment. The larvae were dissected in a fixing solution containing 4% formaldehyde and 1% glutaraldehyde (4F:1G) in 0.1 M sodium phosphate (pH 7.2) to preserve the fine midgut structure during dissection. The Leica M205C stereo microscope (Heerbrugg, Switzerland) was utilized to isolate the midgut for further histopathological and ultrastructural examinations.

Midgut *S. frugiperda* larval tissues from the control and the Btk-treated groups were quickly preserved in 10% buffered formalin for 24 h after dissection to prepare for histology. The samples were dehydrated by subjecting them to increased concentrations of ethyl alcohol (70%, 80%, 90%, and 100% for 15 min each). Afterward, the tissues were embedded in a mixture of Epon and Araldite (Sigma-Aldrich, France). Semithin sections with a thickness of 0.5 µm were obtained by cutting them with an LKB ultramicrotome (model 2088 ultramicrotome, LKB Bromma, USA). After mounting the sections on a microscopic slide, they were stained with 1% water-based toluidine blue and examined under a light microscope (Olympus, model CX31, Japan) to determine any alterations in the structure and organization of midgut cells. Pictures were captured using this light microscope.

### Transmission electron microscopy (TEM) analyses

The midguts from the control and Btk-treated groups were extracted and preserved in 4F:1G buffer within 0.1 M phosphate buffer for 3 h at 4°C. Following immersion in a 2% osmic acid solution, fixation lasted for 2 h at 4°C. The samples were washed in the same solution and then dehydrated at 4°C using a series of increasing ethanol concentrations (from 70 to 99%). Subsequently, the samples were inserted into labeled capsules filled with a mixture of Epon-Araldite. Subsequently, tissues were sliced into ultrathin sections 70 nm thick on copper grids with a mesh size of 200 (Shonouda and Osman 2018). Ultrathin sections were stained with uranyl acetate and lead citrate for 30 min each. They were examined using a transmission electron microscope (TEM) (JEM-1400 Plus, Japan). TEM was utilized with an accelerating voltage of 80 kV to capture electron micrographs at various magnifications.

### Statistical procedures

Using the Origin Pro software system, version 2016 (OriginLab Corporation, Northampton, MA, USA), the concentration-mortality curve and the time-mortality data were estimated. The Kaplan-Meier method was used to analyze survival curves, and the significance of differences between the two groups was recorded with the log-rank test (Plata-Rueda et al. 2022). All genotoxicity and gene expression results were expressed as means with standard errors. SPSS analysis software program Version 20.0 (New York, Armonk: IBM Corp.) was used for all statistical analyses. For normally distributed quantitative variables, a one-way ANOVA test was employed, and the significant differences were then ascertained using a post hoc test (Tukey). The significance of the results was assessed at a 5% level.

## Results

### Concentration‑mortality bioassay on *S. frugiperda* larvae

The concentration-mortality bioassay demonstrated that Btk was toxic to *S. frugiperda* at LC_50_/48 h = 109 µg/100 mL (106–112 µg/100 mL), while no mortality was observed in the control group (Table 2).

**Table 2:** Lethal concentrations of *Bacillus thuringeinsis* var. *kurstaki* (Btk) against *Spodoptera frugiperda* larvae after 48 h of treatment

Number of larvae	Lethal concentrations	Estimated concentrations (µg/100 mL)	Confidence interval 95% (µg/100 mL)	*χ*^2^ (*p* value)
100	LC_25_	95	92 −97	9.977 (0.019*)
	LC_50_	109	106–112	
	LC_75_	127	123–132	
	LC_90_	141	135–146	

The chi-square (*χ*^2^) values refer to the goodness of fit test at *p* ≤ 0.05

### Time‑mortality bioassay on *S. frugiperda* larvae

The survival percentage of *S. frugiperda* larvae treated with different lethal concentrations of Btk for 48 h showed a significant difference (log-rank test *χ*^2^ = 24.619, d.f. = 4, *p* < 0.001), decreasing from 100% in the control group to 78% (LC_25_/48 h), 48% (LC_50_/48 h), 27% (LC_75_/48 h), and 11% (LC_90_/48 h) using the Kaplan-Meier survival analysis (Supplemental Figure 1).

### Effects of Btk on micronuclei formation

**Fig. 1:**
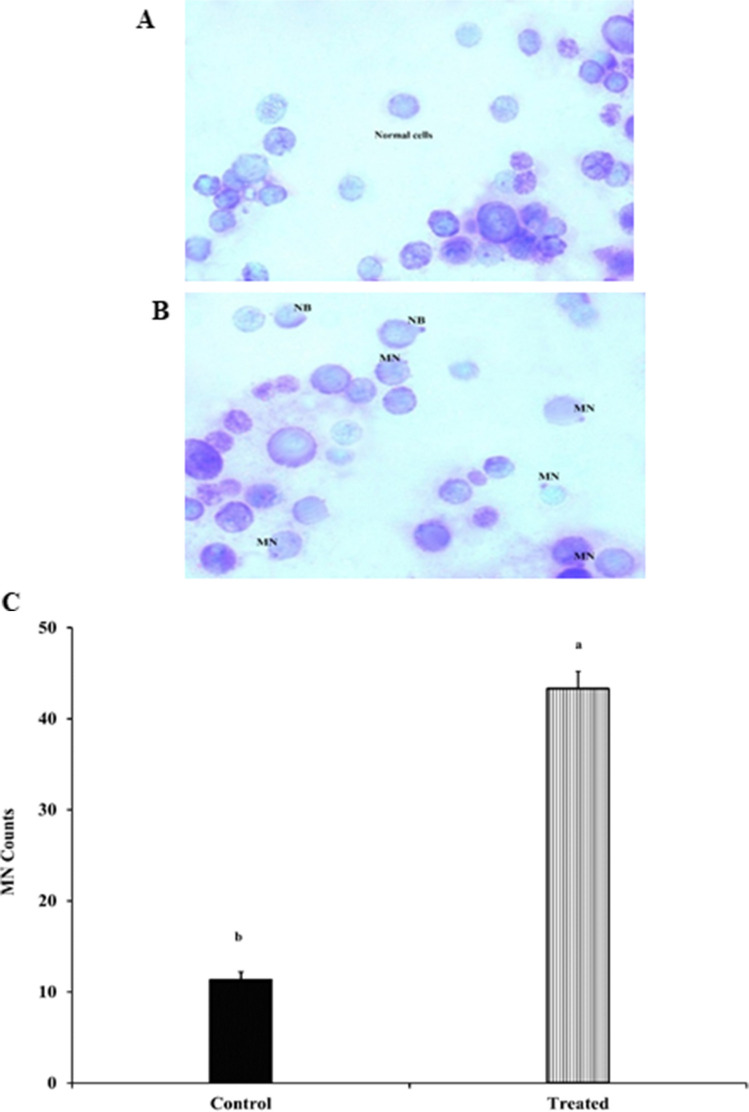
Effect of *Bacillus thuringiensis* var. *kurstaki* (Btk) treatment (LC_25_) on micronuclei formation in the midgut cells of third larval instars of *S. frugiperda*. **A** The normal midgut cells of *S. frugiperda* larvae after 48 h. **B** The incidence of micronuclei (MN) and nuclear bud (NB) in the midgut cells of *S. frugiperda* larvae fed castor leaves treated by Btk after 48 h. Magnification: ×100. **C** Mean ± SE of micronuclei formation in isolated *S. frugiperda* cells. Means followed by the different letters of the alphabet are significantly different according to Tukey’s test at *p* ≤ 0.001 level

Figure 1A and B show the micronuclei formation in the midgut cells of *S. frugiperda* after 48 h due to the Btk LC_25_ effect. The midgut cells of *S. frugiperda* larvae in the control group showed intact nuclei (Fig. 1A). On the other hand, the midgut cells of the Btk-treated group showed visible micronuclei and nuclear buds (Fig. 1B). After 48 h, the mean number of micronucleated cells in the Btk-treated group was significantly higher (43.3 ± 1.9) than that of the control group (11.3 ± 0.88) (F = 518.57, d.f. = 4, *p* < 0.001) (Fig. 1C).

### Effects of Btk on cell viability

**Fig. 2:**
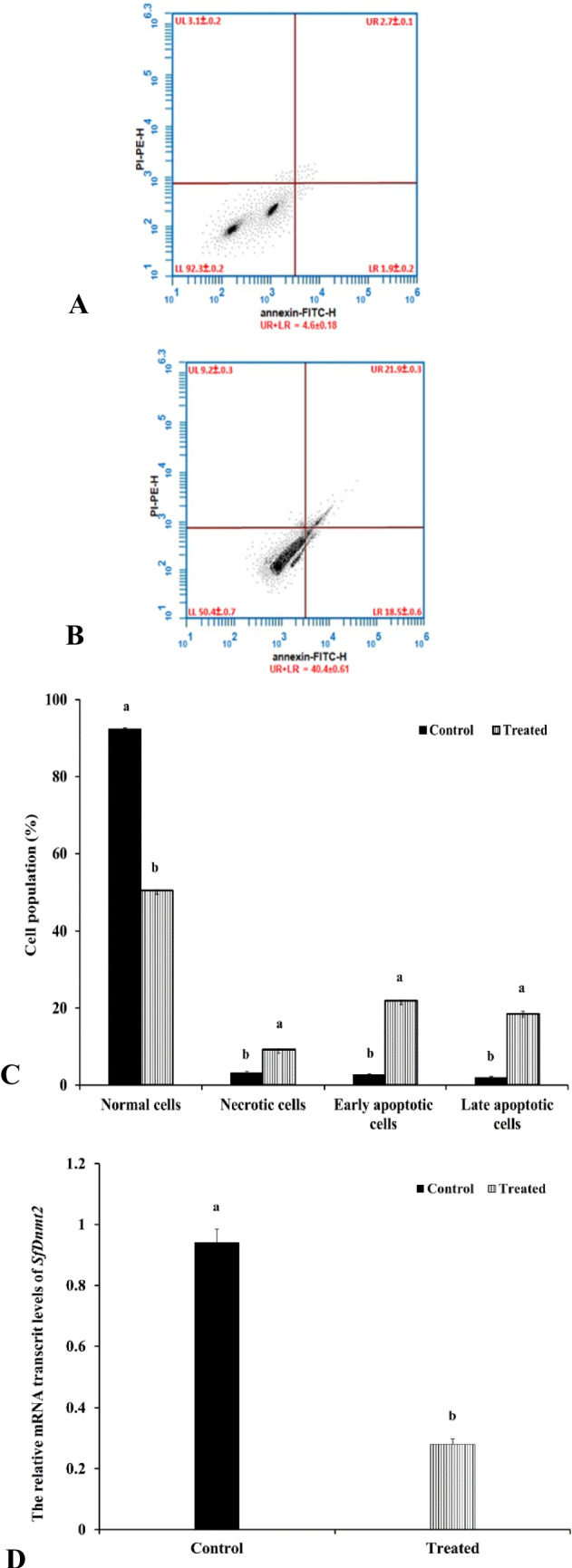
Apoptosis investigation by using annexin V-FITC/PI-staining flow cytometric of the midgut cells of *S. frugiperda* larvae after *Bacillus thuringiensis* var. *kurstaki* (Btk) treatment (LC_25_). **A** The cells from the control group after 48 h. **B** The cells from treated larvae with Btk after 48 h. **C** Mean ± SE of normal, necrotic, and apoptotic cells (early and late) in *S. frugiperda* larval midgut cells. Means followed by different letters of the alphabet are significantly different according to Tukey's test at *p* ≤ 0.001 level. **D** RT-qPCR measurement of *S. frugiperda* DNA methyl transferase 2 (*SfDnmt2*) mRNA expression levels in the midgut cells of *S. frugiperda* treated with LC_25_ of *Bacillus thuringiensis* var. *kurstaki* (Btk). Bars with different letters indicate a significant difference from each other at *p* ≤ 0.05 based on Tukey’s test. Abbreviations: UL, the upper left quadrant corresponds to (PI+/Annexin V−) represents necrotic cells; LL, the left lower quadrant corresponds to (PI−/Annexin V−) represents healthy cells; UR, the upper right quadrant corresponds to (PI+/Annexin V+) represents early apoptotic cells; LR, the lower right quadrant corresponds to (PI−/Annexin V+) represents late apoptotic cells

To evaluate the potential of LC_25_ of Btk to cause apoptosis and necrosis, the midgut cells of *S. frugiperda* larvae were investigated using flow cytometry (annexin V-FITC assay) after 48 h of treatment (Fig. 2A, B). Normal cells are shown in the lower left quadrant, necrotic cells in the upper left quadrant, early apoptotic cells in the upper right quadrant, and late apoptotic cells in the lower right quadrant. After 48 h of Btk treatment, the number of normal cells declined sharply to 50.37 ± 0.67 (*F* = 14,850.5, d.f. = 4, *p* < 0.001). In contrast, the number of necrotic cells increased significantly to 9.2 ± 0.32, compared to the control group which showed 92.3 ± 0.21 normal cells and 3.1 ± 0.20 necrotic cells (*F* = 185.11, d.f. = 4, *p* < 0.001) (Fig. 2C).

The mean values of early and late apoptotic midgut cells were significantly higher in the Btk-treated group (21.9 ± 0.32 and 18.5 ± 0.64, respectively) than in the control group. The mean values of early and late apoptotic cells in the control group were 2.7 ± 0.15 and 1.9 ± 0.17, respectively (early: *F* = 1109.84, late: *F* = 1876.54, d.f. = 4, *p* < 0.001).

### Effects of Btk on the expression level of SfDnmt2

To determine the effects of Btk (LC_25_) on the *SfDnmt2* gene, the present study analyzed the expression level using real-time PCR (RT-qPCR) in the larval midgut cells (Fig. 2D). The results indicated a slight downregulation of *SfDnmt2* mRNA expression in response to Btk treatment for 48 h (0.28 ± 0.02), which was significantly lower than the control group (0.94 ± 0.04) (*F* = 206.51, d.f. = 4, *p* < 0.001).

### Cytotoxicity, apoptosis, and necrosis of BtK-treated larval midgut cells

**Fig. 3:**
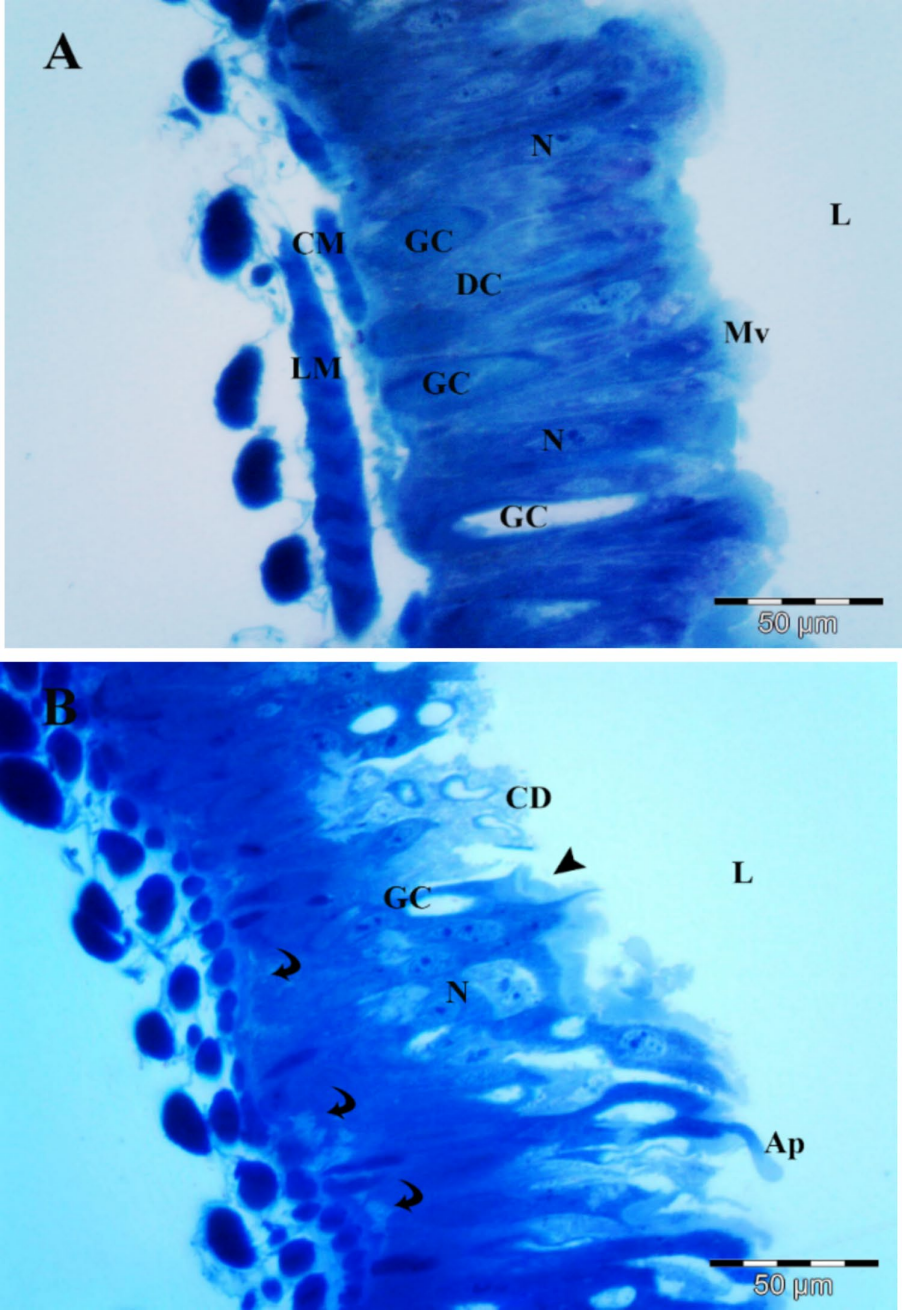
Histological section of the midgut of 3^rd^ *S. frugiperda* larvae. **A** Control group showing the midgut epithelium based on external longitudinal (LM) and internal circular (CM) muscle layers, regular digestive cells (DC) with nucleus (N), goblet cells (GC), and well-developed apical microvilli (Mv) in the lumen (L). **B** *Bacillus thuringeinsis* var. *kurstaki* (Btk)–treated group (LC_25_) with disorganized epithelium rich in apical cell debris (CD), reduced goblet cell (GC), cytoplasmic space (black curved arrow), degraded microvilli (black arrowhead), and apocrine secretion (Ap) in the lumen (L)

The toxic action of Btk can trigger histolysis of midgut tissues, potentially contributing to increased larval mortality. To confirm this, we examined the *S. frugiperda* midguts histologically. The epithelium of the control *S. frugiperda* larval midgut consisted of goblet cells, high columnar digestive cells, and a noticeable peritrophic matrix (Fig. 3A). The medial-basal part of the cells contained an elongated nucleus primarily composed of decondensed chromatin.

Forty-eight hours after treatment with LC_25_ of Btk, *Spodoptera frugiperda* larvae showed histological alterations in their midgut (Fig. 3B). Cellular degeneration, abnormal cell shapes, cell fragments, and apocrine secretions began to appear in the lumen. The peritrophic matrix had ruptured, and the cytoplasm was highly vacuolized.

### Ultrastructural alterations of the midgut of *S. frugiperda*

**Fig. 4:**
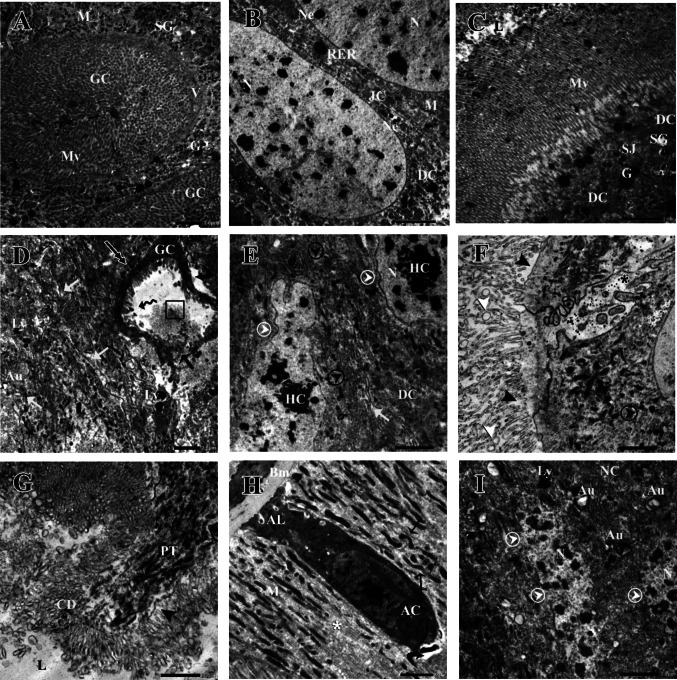
Transmission electron micrographs of goblet cells and digestive cells from the control (**A**–**C**) and treated (**D**–**I**) midgut of the 3^rd^ larval instar of *S. frugiperda*. **A** Goblet cell (GC) manifested intact valve (V) with well-developed microvilli (Mv), secretory granules (SG), large electron-dense granules (G), and abundant mitochondria (M). **B** The basal region of two adjacent digestive cells (DC) connected by intact junction complex (JC) with nucleus (N), regular nuclear envelope (Ne), and perinuclear cytoplasm associated with mitochondria (M). **C** The apical region of digestive cells with well-organized microvilli (Mv) and tight and intact the septate junction (SJ). **D** Goblet cells (GC) had a distorted shape with damaged microvilli (black wavy arrow), cytoplasmic space (black curved arrow), microvilli fragments inside the cavity of goblet cell (black square), the disintegrated junction complex (white arrow), and the presence of lysosomes (Ly), the accumulation of mitochondria around the goblet cell (two-headed arrow), the absence of goblet cell valve (black arrow), and the presence of autophagic vacuoles (Au). **E** The basal region of digestive cells (DC) exhibited heterochromatin (HC), which was distributed within the nucleus (N) and along the irregular nuclear envelope (white circle arrowhead) and fragmented rough endoplasmic reticulum (black circle arrowhead). **F** The apical region of digestive cells (DC) with completely damaged microvilli (black arrowhead), microapocrine vesicles (white arrowhead) pinching off from the microvilli and the uncoupled septate junction (black asterisk). **G** The protrusion formation (PT) at the apical region of digestive cells and cell debris (CD) released to the lumen (L). **H** Apoptotic digestive cell formation (AC) filled with agglomerated chromatin and autolysosomes (AL) based on the basement membrane (Bm), scant cytoplasm (white asterisk), numerous mitochondria (M), and nanotunnel mitochondria formation (black spear arrow). **I** Necrotic digestive cell (NC) with complete lysis of its nucleus (N) and nuclear envelope (white circle arrowhead) and numbers of autophagic vacuoles (Au)

In Fig. 4A–C, the midgut of control *S. frugiperda* larvae had a single epithelial layer of columnar digestive cells organized alternately with goblet cells, as revealed by transmission electron microscopy (TEM). The goblet cells were round or oval, possessed an intact valve, and had abundant cytoplasmic projections (microvilli) in their internal cavities (Fig. 4A). Additionally, in the cytoplasm of the midgut, numerous mitochondria, large dense granules, and secretory granules were found. The basal part of the digestive cell has nuclei with decondensed chromatin and intact nuclear envelopes. Well-developed rough endoplasmic reticulum (RER) was visible near the junction between the cells (Fig. 4B). RER cisternae were arranged in parallel arrays and the septate junction was well-organized and connected between two adjacent digestive cells (Fig. 4B). At the apical part of the digestive cell, the brush border of the peritrophic matrix facing the midgut lumen was made of numerous structured microvilli with consistent thicknesses and lengths (Fig. 4C).

The goblet and columnar digestive cells of the *S. frugiperda* larval midgut showed remarkably deteriorated ultrastructural alterations after 48 h of treatment with LC_25_ of Btk (Fig. 4D–I). The goblet cell had a distorted shape, its projections (microvilli) were damaged, and microvilli debris could be detected inside the goblet cell cavity (Fig. 4D). An aberrant goblet cell feature was the loss of its valve and disorderly arrangement of its constituents, along with an abnormal accumulation of mitochondria surrounding them (Fig. 4D). Furthermore, the intercellular spaces were mostly widened, lysosomes and autophagic vacuoles were observed, and prominent destructive changes occurred in the intercellular junction complex (Fig. 4D).

The progressive impacts of the Btk delta-endotoxin on *S. frugiperda* midgut epithelial cells resulted in irregular and elongated digestive cells. The nucleus in the basal cell portion of the digestive cell had condensed heterochromatin clusters with the asymmetrical nuclear envelope (Fig. 4E). Moreover, Btk treatment caused a partial depletion of the cytoplasm and RER fragmentation (Fig. 4E). Also, the microvilli were scattered around the cells and showed irregularities at the apical regions of the digestive cells (Fig.  4 F). Likewise, microapocrine vesicles seemed to pinch off from the microvilli’s apex, and the septate junction separated and detached. Eventually, remarkable damage to digestive cells was detected causing cytoplasmic content to leak out and release cell debris into the lumen forming an apical protrusion (Fig. 4G).

Btk intoxication led to the observation that the heterochromatin fractured inside an apoptotic digestive cell, along with autolysosomes and scant cytoplasm, and numerous nanotunnel mitochondria, serving as a cytotoxic criterion (Fig. 4H). Additionally, the cells underwent necrosis 48 h after Btk treatment with complete lysis of their nucleus and nuclear envelope (Fig. 4I).

## Discussion

Most studies utilize acute lethal toxicity to control diverse insect pests without clarifying its effect on various biological processes. In contrast, studying the effects of sublethal concentrations could elucidate the impact on various biological parameters of insect pests. Therefore, the present study focused on understanding the toxic impact of sublethal concentration treatment with microbial Bt on the cytogenetic alterations of the maize insect pest *S. frugiperda* larvae.

This study demonstrated that Btk exhibited pronounced mortality and toxicity on *S. frugiperda* larvae after 48 h of feeding on treated leaves. The Bt toxicity on *S. frugiperda* larvae caused an increase in mortality percentages and the notable detrimental effects on larval survival and growth, consistent with findings by Polanczyk et al. (2000) and Lima et al. (2010). This aligns with results observed in other lepidopteran pests treated with Btk, such as *S. litura* (Vineela et al. 2016) and *Helicoverpa armigera* (Kuss et al. 2016). The mortality percentages are attributed to toxins of Btk’s action. These toxins bind to midgut epithelial cells, creating pores in the cell membranes. Consequently, midgut motility is impaired due to the lysis of epithelial cells, and the larvae cease feeding. Bacterial spores can germinate intensively and penetrate host tissue, resulting in septicemia and insect death (Ren et al. 2002).

Regarding larval treatment with the Btk sublethal concentration, it was noted that the development of surviving larvae was delayed due to reduced food consumption, and they did not complete their development. This indicates that using sublethal concentrations can be a beneficial strategy for controlling *S. frugiperda* within an integrated pest management program. The usage of sublethal concentration is an explanatory study that is rarely considered when evaluating the efficacy of biological agents.

Little information was available on the genotoxic effects of Btk sublethal concentration on *S. frugiperda* larvae. In the present study, a statistically significant difference in mean micronucleus (MN) formation values between the control and Btk-treated groups was observed in the third-instar *S. frugiperda* larval midgut after 48 h. MN is the predominant biomarker for assessing the effects of environmental contaminants and chemicals on insect populations (Luzhna et al. 2013). In midgut cells, the Bt toxins enhanced MN formation, indicating their genotoxicity (Grisolia et al. 2009). MN manifests as a tiny nucleus adjacent to the main nucleus. This small nucleus arises from a lagging chromosome or an acentric fragment of chromatin that fails to be incorporated into the main nucleus during cell division (Ahmadi et al. 2015). Notably, the development of MN and nuclear buds indicated genomic instability, which may lead to decreased cell vitality (Santovito et al. 2020). According to Hintzsche et al. (2017), the overall occurrence of micronuclei appeared to serve as an apoptotic signal. A similar pattern of genotoxic response, characterized by micronuclei formation, was observed when *Lycaena dispar* (Lepidoptera: Lycaenidae) and *S. frugiperda* larvae were treated with herbicide and albendazole, respectively (Santovito et al. 2020; Malak 2023).

Apoptosis or necrosis can result from severe DNA damage and fragmentation. Annexin V assay is recommended as a valuable indicator of apoptosis. It can be a recombinant phosphatidylserine-binding protein that interacts precisely with the phosphatidylserine residues of damaged cells, a characteristic feature of later stages of apoptosis or necrosis (Elmore 2007). Therefore, the annexin V assay used in the present study confirmed that the LC_25_ of Btk induced both apoptosis and necrosis processes in *S. frugiperda* larval midgut cells. After 48 h of Btk treatment, a significant increase in apoptotic and necrotic cells was observed. The apoptotic cells are characterized by chromatin condensation, DNA fragmentation, and cellular shrinkage. On the other hand, necrotic cells appeared to be enlarged and lysed. Our findings coincided with the results of Bt-treated lepidopteran pest larvae reported by Loeb et al. (2000) in *Heliothis virescens* and Chauhan et al. (2017) in *Achaea janata*, respectively.

The gene expression analysis of *SfDnmt2* in the larval midgut cells in this study revealed that, after 48 h of treatment with the LC_25_ of Btk, there was a significant decrease in *SfDnmt2* activity. Bacterial infections can change the DNA methylation state of insects, potentially changing gene expression and impacting various physiological processes, particularly immunity (Kausar et al. 2022b). DNMT contributes to the interactions between insects and pathogens, as evidenced by the consequences of bacterial infections and the alteration of the immune response (Kausar et al. 2022a). Furthermore, the evidence suggests that the suppression of the DNMT gene expression appears to be a viable molecular strategy for controlling insect pests, which affects the survival of insects and triggers apoptosis (Kausar et al. 2021). The present results for *SfDnmt2* are consistent with the findings of Baradaran et al. (2019) and Kausar et al. (2022a), who reported that RT-qPCR assays showed an abrupt decrease in *Dnmt2* expression levels at 48 h of Bt treatment in *Helicoverpa armigera* and *Antheraea pernyi*, respectively.

Using light and transmission electron micrographs, this study examined the morphological and ultrastructural alterations of goblet and columnar digestive cells in third-instar *S. frugiperda* larvae fed the LC_25_ of Btk for 48 h. Proteolytic processing, binding to specific receptors, and pore formation are the action mechanisms of Btk toxins (Sousa et al. 2010). Due to their highly rapid mode of action, Btk toxins drastically alter and nearly destroy the midgut environment within 48 h of feeding, as the midgut epithelium is their primary target.

Therefore, the evaluation of detrimental effects focused on the midgut cells of *S. frugiperda* larvae treated with Btk, compared to control larvae. Goblet cells exhibited notable morphological changes. Their shapes were deformed, their plasma membrane projections (microvilli), which define the goblet cavities, were reduced or absent, and their valves were absent following the LC_25_ of Btk feeding. The primary energy source in the goblet cells is the enzyme V-ATPase, which also functions as an ionic transporter of H^+^/K^+^ across insect midguts (Sousa et al. 2010) and serves as a binding site for the Bt toxin (Krishnamoorthy et al. 2007). Similarly, the goblet cells of *H. armigera* showed alterations following Bt treatment (Abd El-Ghany et al. 2015) and *S. frugiperda* after treatment with anthelmintic drugs (Malak et al. 2025). This demonstrates that the goblet cells are the primary site of the toxin action; however, secondary effects through the columnar cells cannot be excluded (Pandey et al. 2009).

Abnormalities in columnar digestive cells, mainly caused by Btk treatment, were irregularly shaped and disorganized in the midgut, as well as nucleus shrinkage, nuclear chromatin condensation, nuclear envelope destruction, and RER destruction. Daquila et al. (2019) reported that Bt toxic action may be the cause of columnar cell damage in the larval midgut. Furthermore, the presence of lysosomes was linked to the cytoplasmic spaces as a principal cytotoxic response. The presence of cytoplasmic spaces triggered the autophagy process in midgut cells caused by Bt (Tewfick and Soliman 2018). Comparably, Castro et al. (2019) observed columnar cells exhibited histological and ultrastructural damage with Bt treatment in *Anticarsia gemmatalis*.

Furthermore, toxicological criteria were observed following Btk treatment, and the *S. frugiperda* midgut peritrophic matrix was ruptured. The peritrophic matrix is essential for absorbing nutrients in the digestion process. Moreover, it delays contact with digestive cells by shielding the epithelial cells from the introduction of Btk toxins. However, these toxins can enter the peritrophic matrix, attach to the columnar microvilli’s receptors (Castro et al. 2019), and infect the midgut cells of *S. frugiperda*. Moreover, Btk action on the cytoskeleton actin showed the degradation of microvilli in *S. frugiperda* digestive cells. Additionally, the Btk treatment led to separated cells at the intercellular junctional complex and the septate junction. The lack of tissue cytoskeleton resulted in a loss of membrane integrity (Feng et al. 2014). Furthermore, the outer midgut of treated larvae showed secreted apocrine and microapocrine vesicles. Btk increased the secretion of microvesicles as a detoxification mechanism, most likely to prevent or counteract the infection as reported in *A. gemmatalis* larvae when treated with nucleopolyhedrovirus (Levy et al. 2011).

In the present study, the Btk toxins affected the midgut cell membrane structure, leading to cytoplasm leakage into the midgut lumen. It is interesting to note that *S. frugiperda* larvae fed on LC_25_ of Btk released cellular protrusions into the midgut lumen. The damage to midgut epithelial cells following Btk consumption may indicate a cytotoxic effect and a detoxifying response. This damage could then lead to apoptosis. Apoptosis is important for tissue homeostasis, healthy cell division, and elimination of undesirable cells, particularly those resulting from bacterial or physical damage. Apoptotic cells exhibited morphological features such as DNA fragmentation and the formation of apoptotic bodies (Jiang et al. 2016). These results confirmed that treatment with a sublethal concentration of Btk triggered apoptotic pathways in the *S. frugiperda* midgut. Both intrinsic and extrinsic pathways can cause apoptosis. According to Zou et al. (2024), *Bombyx mori* became poisoned by low concentrations of Bt, potentially when the apoptotic process was triggered.

Interestingly, the midgut treated with LC_25_ of Btk showed structural alterations by forming nanotunnel mitochondria. Fiaz et al. (2018) reported that the nanotunnels could be a sign of moderately compromised respiratory chain function in the mitochondria (Vincent et al. 2016). These mitochondrial alterations could be attributed to the oxidative stress of the Bt sublethal concentration treatment, leading to the induction of autophagy and apoptosis (Farder-Gomes et al. 2021). Consequently, autophagic vacuoles were found in the midgut of *S. frugiperda* larvae treated with LC_25_ of Btk, suggesting that the cells could undergo cytoplasmic reorganization. Autophagic vacuoles play a vital role in maintaining homeostasis and mobilizing cellular energy by eliminating pathogens and damaged organelles (Fiaz et al. 2018). Furthermore, oxidative stress may be related to the accumulation of mitochondria with various shapes in Btk-treated larvae. Mitochondria play a crucial part in how the insect body responds to bacterial infection by providing energy in the form of ATP.

Additionally, Btk caused necrosis in the midgut cells of *S. frugiperda* and significantly impaired and degraded the epithelial cells. Lysosomes seem to be a pivotal factor in the progressive necrosis that developed in the midgut epithelia (Poopathi et al. 2008). The destruction and necrosis of epithelial cells were histopathological alterations observed in the larval midgut of *S littoralis* treated with *Bacillus* sp. (Ghribi et al. 2012). *Heliotis virescens* larvae died after 48 h of exposure to Bt due to membrane damage and necrosis (Loeb et al. 2000). Our results demonstrated that *S. frugiperda* larvae had a higher sensitivity to Btk, and the sublethal concentration was sufficient to produce adverse effects, suggesting that Btk is a highly effective method for controlling *S. frugiperda* larvae.

In conclusion, the present study provided additional valuable information on the cellular toxic action of Btk sublethal concentrations. The results indicated that the sublethal concentration of Btk reduces the survival percentage of *S. frugiperda* pest. Interestingly, the genotoxicity examination, along with histopathological studies, revealed the post-treatment toxic effects of Btk sublethal concentration. Moreover, the results showed that apoptosis and necrosis processes were activated after LC_25_ of Btk treatment, leading to severe damage to the midgut cells. The promising results in the laboratory encourage further studies in the field to evaluate the potentially toxic effects of Btk sublethal concentration against the economically destructive *S. frugiperda* pest. It could be used as a successful strategy in the IPM program for controlling *S. frugiperda* pest.

## Supplementary Information

Below is the link to the electronic supplementary material.

ESM1 (DOCX 127 KB)

## Data Availability

The datasets utilized and analyzed during this investigation are available upon reasonable request from the corresponding author.
