# Supplementary material for: Bacillus thuringiensis Sublethal Concentration Effectively Alter Cytogenetic Response of Spodoptera frugiperda Larvae
Source: Neotrop Entomol. 2026 Apr 16;55(1):32. doi: 10.1007/s13744-026-01384-9 (PMC13086812; doi:10.1007/s13744-026-01384-9)
Supplement: Supplementary file 1 — (DOCX 127 KB) [file 13744_2026_1384_MOESM1_ESM.docx]

***Bacillus thuringiensis* Sublethal Concentration Effectively Alter Cytogenetic Response of *Spodoptera* *frugiperda* larvae**

**Marian Malak^a^*, Mourad Shonouda^b^**

**Supplemental Table 1**

***Spodoptera frugiperda* larval mortality percentage after 48 hours of treatment by Btk diluted by ddH_2_O**

| **Btk Concentrations**  **(µg/100 mL)** | **Larval mortality percentage**  **(%)** |
| --- | --- |
| 150 µg | 100 |
| 140 µg (LC_90_) | 89 |
| 130 µg (LC_75_) | 78 |
| 120 µg | 66 |
| 110 µg (LC_50_) | 53 |
| 100 µg | 39 |
| 95 µg (LC_25_) | 22 |
| 90 µg | 13 |
| 85 µg | 3 |

**Supplemental Figure (1)**


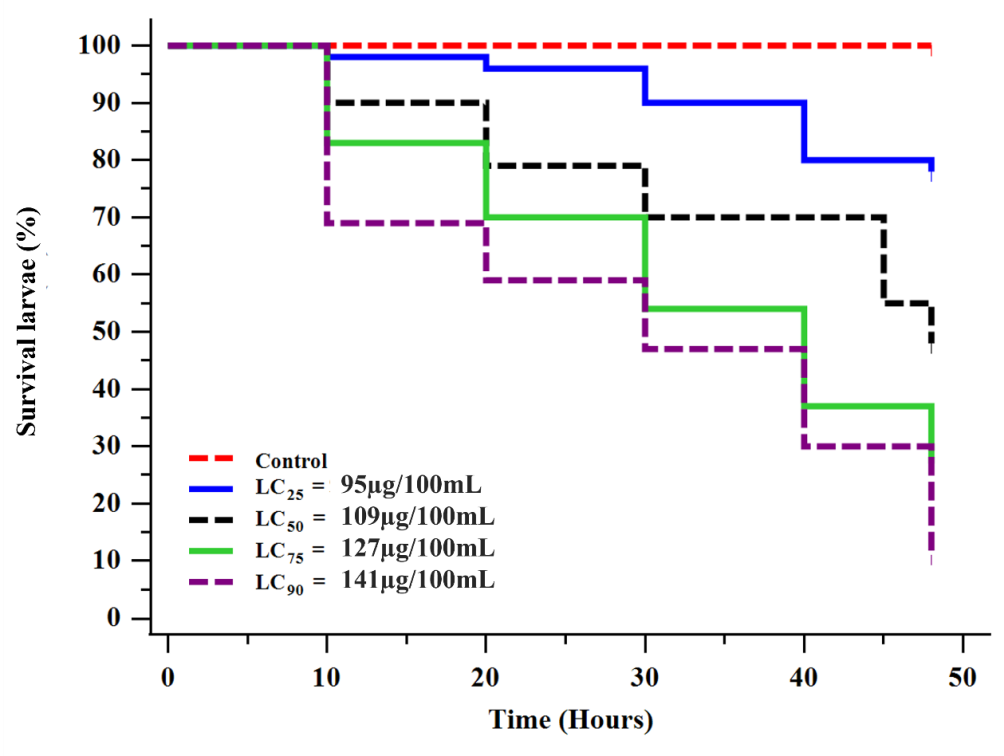


**Figure (1)** Survival curves of *Spodoptera frugiperda* larvae treated with different lethal concentrations (LC) of *Bacillus thuringeinsis* var. *kurstaki* (Btk), subjected to survival analysis using the Kaplan–Meier analysis (log-rank test χ^2^ = 24.619; *P* < 0.001)
